# A cross sectional study assessing steatotic liver disease in patients with systemic lupus erythematosus

**DOI:** 10.1038/s41598-024-65105-1

**Published:** 2024-06-20

**Authors:** Armando Antonio Baeza-Zapata, Ashuin Kammar-García, Ana Barrera-Vargas, Javier Merayo-Chalico, Sophia Eugenia Martínez-Vázquez, Carlos Moctezuma-Velazquez

**Affiliations:** 1 Gastroenterology Department, Instituto Nacional de Ciencias Médicas y Nutrición Salvador Zubirán, Vasco de Quiroga 15, Colonia Belisario Domínguez Sección XVI, Tlalpan, CP 14080 Mexico City, Mexico; 2 Research Division, Instituto Nacional de Geriatría, Av Contreras 428, San Jerónimo Lídice, Magdalena Contreras, CP 10200 Mexico City, Mexico; 3 Immunology and Rheumatology Department, Instituto Nacional de Ciencias Médicas y Nutrición Salvador Zubirán, Vasco de Quiroga 15, Colonia Belisario Domínguez Sección XVI, Tlalpan, CP 14080 Mexico City, Mexico; 4 Division of Gastroenterology (Liver Unit), Zeidler Ledcor Centre, University of Alberta, 8540 112 Street NW, Room 1-20B, Edmonton, AB T6G 2X8 Canada

**Keywords:** Fatty liver, Immune system disease, Lupus erythematosus, systemic, Hydroxychloroquine, Metabolic syndrome, Liver, Hepatology, Non-alcoholic fatty liver disease

## Abstract

Patients with immune-mediated inflammatory diseases are prone to steatotic liver disease (SLD), which has been observed in patients with psoriasis and hidradenitis suppurativa. We aimed to assess whether systemic lupus erythematosus (SLE) was associated with SLD and to define factors associated with SLD in SLE. This was a cross-sectional study, we included 106 consecutive patients with SLE who were seen in the rheumatology clinic between June 2021 and March 2022 and we chose two sex-paired controls for each SLE. All the participants underwent FibroScan and anthropometric assessments. SLD was defined as a controlled attenuation parameter ≥ 275dB/m. Prevalence of SLD was lower in patients with SLE (21.7% vs 41.5%, p < 0.001). Patients with SLE and SLD had a lower frequency of hydroxychloroquine use (65% vs 84%, p = 0.04), and higher C3 levels [123mg/dl (IQR 102–136) vs 99mg/dl (IQR 78–121), p = 0.004]. Factors associated with SLD in SLE were body mass index (BMI), waist circumference, glucose, and C3; hydroxychloroquine use was a protective factor. On univariate analysis, SLE was associated with a reduced risk of SLD (OR 0.39, 95%CI 0.23–0.67); however, after adjusting for age, BMI, waist, glucose, triglycerides, high-density cholesterol, low-density cholesterol, leukocytes, and hydroxychloroquine, it was no longer associated (OR 0.43, 95%CI 0.10–1.91). In conclusion, the prevalence of SLD in patients with SLE was not higher than that in the general population, and SLE was not associated with SLD. The factors associated with SLD were anthropometric data, glucose, hydroxychloroquine, and C3 levels.

## Introduction

Immune-mediated inflammatory diseases (IMIDs) are a group of diseases in which the immune system is significantly dysregulated. IMIDs are characterized by persistent chronic inflammation thought to be due to an imbalance in the production of cytokines and adipokines^1^. They can be found in 5 to 7% of the general population^2^. Due to the state of chronic inflammation, some patients with IMIDs are prone to developing steatotic liver disease (SLD) along with the other components of metabolic syndrome, explaining at least in part the higher rate of cardiovascular disease that we see in these patients^3^. When compared with the general population, a higher prevalence of SLD has been observed in patients with psoriasis, hidradenitis suppurativa, and inflammatory bowel disease^4–6^. In contrast, a recent meta-analysis in patients with rheumatoid arthritis found a pooled prevalence of 35%, which is similar to that reported in the general population^7^. This is important because SLD is currently the most common etiology of liver disease worldwide and is associated not only with increased risk of end-stage liver disease and hepatocellular carcinoma, but also of cardiovascular disease, chronic kidney disease, and cancer^8^, suggesting screening would be justified in patients thought to be at increased risk.

Systemic lupus erythematosus (SLE) is the prototype of an autoimmune disease, and as with other IMIDs, is also associated with a higher risk of cardiovascular disease and metabolic syndrome^3,9,10^. Patients with SLE may develop different liver conditions during their disease course, like hepatitis as a manifestation of SLE itself, drug-induced liver injury, SLD, or concomitant autoimmune liver diseases, such as autoimmune hepatitis. In recent years, the most common finding in patients with SLE who undergo liver biopsy has been SLD. In contrast, a previous study of 60 patients with SLE reported a frequency of SLD in line with that of the general population^11^. Therefore, due to the implications of having SLD, this study aimed to assess whether SLE is associated with SLD and to determine which factors are associated with SLD and with significant fibrosis in these patients.

## Methods

### Study design

This was a cross-sectional single-center study in which all consecutive adult patients with known SLE who were seen in a rheumatology clinic in a tertiary referral center in Mexico City between June 2021 and March 2022 were invited to participate. The inclusion criteria were a diagnosis of SLE according to the 2019 European League Against Rheumatism/American College of Rheumatology criteria^12^, and no known underlying liver disease (e.g., autoimmune hepatitis, primary biliary cholangitis, primary sclerosing cholangitis, chronic hepatitis B or C, hereditary hemochromatosis, Wilson´s disease, alcohol-associated liver disease), except for SLD. Patients were also ineligible if they reported drinking more than 20 or 30 g of ethanol/day for female and male participants, respectively. We excluded patients who had not had liver function tests and/or complete blood count in the previous 6 months, or if the liver stiffness measurement by transient elastography (LSM-TE) was technically unsuccessful or unreliable (i.e. IQR/med > 30% and/or less than 10 valid measurements). All procedures followed were in accordance with the Helsinki Declaration of 1964 and its later amendments. In addition, this study was approved by the Research Ethics Committee of the Instituto Nacional de Ciencias Medicas y Nutrición Salvador Zubirán (reference numbers 3770 and 3794). Informed consent was obtained from all patients to participate in the study.

During the study visit an experienced rheumatologist performed a physical examination on each patient and computed the Systemic Lupus Erythematosus Disease Activity Index 2000 (SLEDAI-2K)^13^ and Systemic Lupus International Collaborating Clinics/American College of Rheumatology Damage Index (SLICC/ACR-DI)^14^ scores. Subsequently, patients underwent LSM-TE using the FibroScan 502 (Echosens, Paris, France). Each patient´s records were reviewed to collect clinically relevant information, which included, but was not limited to, the following: age, sex, body mass index (BMI), liver function tests, complete blood count, current and previous medications, and known components of metabolic syndrome (*i.e.* dyslipidemia, prediabetes, diabetes, arterial hypertension). We defined pre-diabetes according to the conventional criteria established by the American Diabetes Association, which includes an HbA1c level between 5.7 and 6.4% and/or fasting plasma glucose levels between 100 and 125 mg/dl^15^. To calculate cumulative corticosteroid use, we added up all periods of corticosteroid treatment, taking into account the particular dose, and expressed it in equivalent mg of prednisone if different steroids were used.

To determine whether SLE was independently associated with SLD, we randomly chose two sex-paired controls from another cross-sectional study conducted at the same time in our center. This cross-sectional study aimed to determine the association between different dietary patterns and SLD in healthcare workers. Patients who were eligible to participate in that study needed to be at least 18 years old, and free of significant comorbidities (i.e. cancer, heart disease, uncontrolled thyroid disease, systemic autoimmune diseases, and chronic kidney disease) other than diabetes, hypertension, overweight, and/or obesity. Diabetes and hypertension had to be at least partially controlled, defined as HbA1c < 8.5% and blood pressure < 160/90mmHg, respectively. Patients who knew beforehand that they had an underlying liver disease, including SLD, were also non-eligible. These patients underwent a clinical examination, including anthropometric assessment, LSM-TE, and a comprehensive lab-work profile, including complete blood count and metabolic parameters.

### Liver stiffness measurement by transient elastography and controlled attenuation parameter

LSM-TE and the controlled attenuation parameter were determined using a FibroScan 502 (Echosens, Paris, France) by an experienced physician (ABZ). For this study, patients fasted for at least three hours. The procedure was performed as described by the manufacturer elsewhere^16^ with the objective of obtaining 10 valid measurements and an IQR/med < 30%. SLD was defined as a controlled attenuation parameter (CAP) ≥ 275dB/m^17^, and a patient was considered to have significant fibrosis when the LSM-TE was ≥ 8.2 kPa^18^.

### Sample size calculation and statistical analysis

Based on reports on the prevalence of SLD in patients with psoriasis (47–52%)^19^, which is another IMID, considering a maximum expected prevalence of 49% in SLE, and 30% in the general population, we would need a sample size of at least 100 patients per group^20^, considering a power of 80% and a 95% confidence level. Numerical variables were summarized with median and interquartile range and categorical variables as frequencies and percentages. The Mann–Whitney U test, χ^2^, and Fisher´s exact test were used to compare characteristics between groups, as appropriate. We conducted logistic regression analysis to assess the factors associated with SLD and significant fibrosis in the entire population and in patients with SLE. Variables with a p-value ≤ 0.1 on univariate analysis were included in the multivariable analysis. Statistical significance was set at p-value < 0.05. Patients with any missing data were excluded from the multivariate analysis. All analyses were conducted using STATA v.14 (StataCorp, Texas, USA), and figures were created using GraphPad Prism v.9.0.3 (GraphPad Prism, San Diego, California, USA). This study was compliant with criteria set out in Strengthening the Reporting of Observational Studies in Epidemiology (STROBE).

## Results

### General characteristics of the population and comparison between patients with SLE and controls

We included 108 patients with SLE and 212 sex-matched controls in our study; we excluded two patients with SLE because their LSM-TE did not satisfy the quality criteria. For the multivariate analysis, we excluded 14 patients with SLE because of missing data (i.e. waist circumference). The median age was 42 years (IQR 30–52), and 267 (84%) participants were female. Although patients with SLE tended to be younger than controls, there was no significant difference in age between the groups (p = 0.07). Median BMI and waist circumference were 26.6 (IQR 23.3–30) and 88 cm (IQR 78.5–96), respectively. Eleven (3.5%) patients had type 2 diabetes, 80 (25.2%) were obese, and 36 (11%) had arterial hypertension.

**Figure 1:**
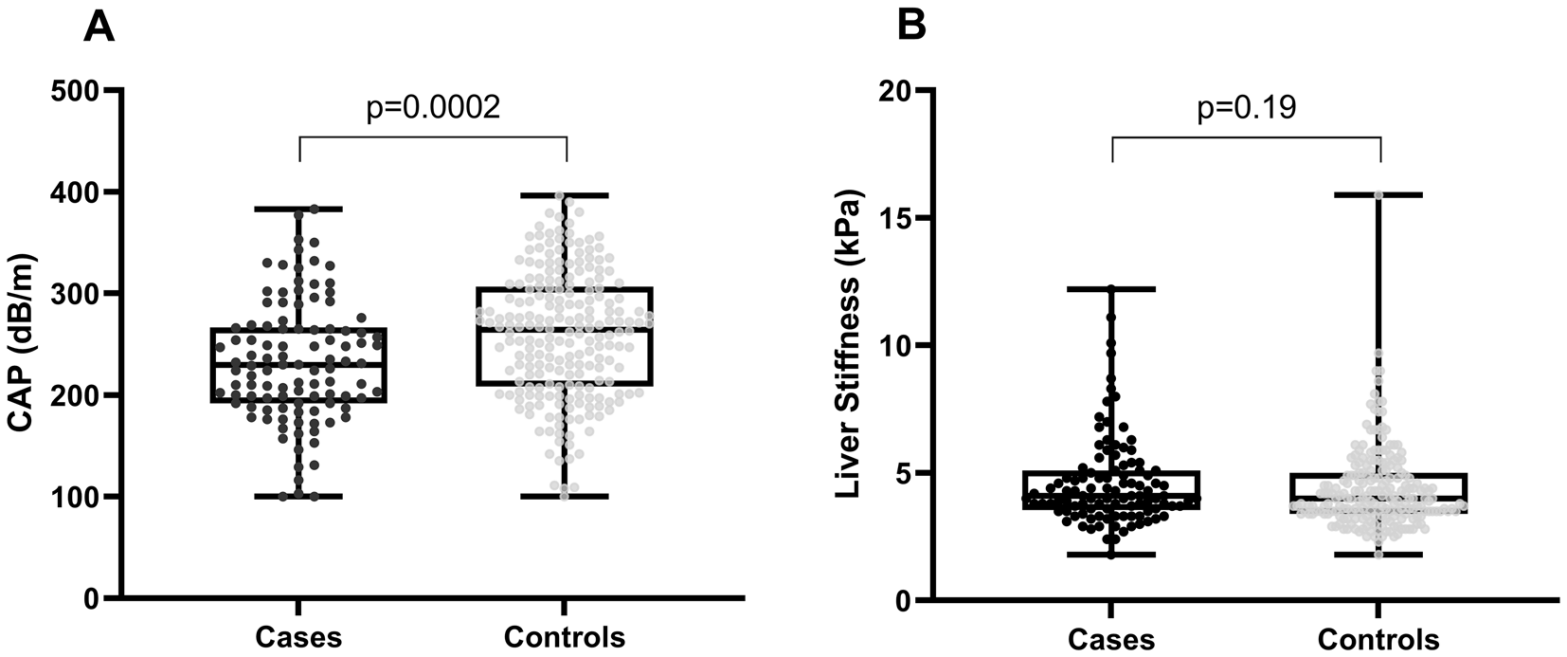
Comparison of FibroScan values between patients with systemic lupus erythematosus (SLE) and controls. (**A**) Controlled attenuation parameter (CAP). (**B**) Liver stiffness.

**Figure 2:**
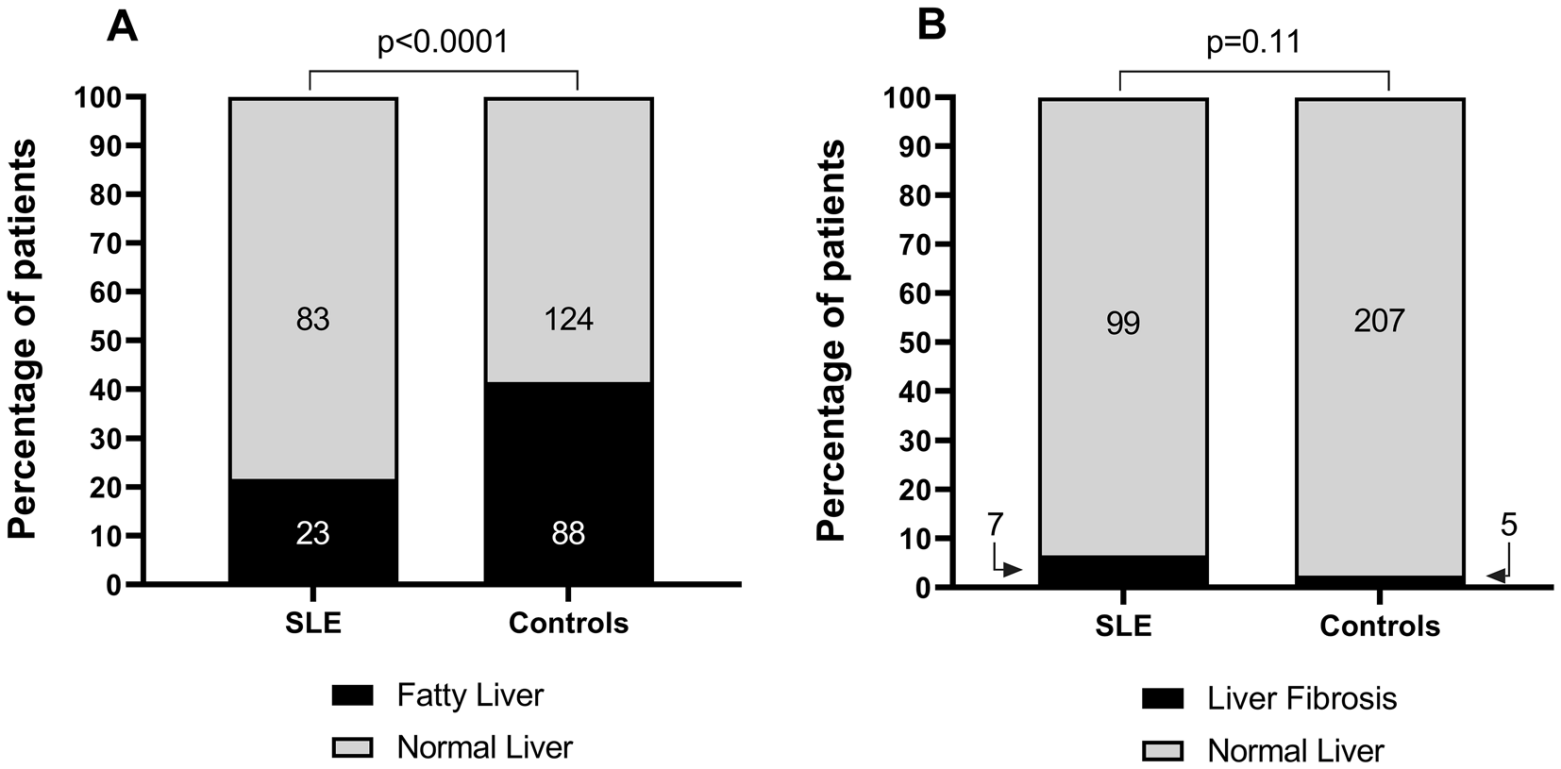
(**A**) Comparison of prevalence of steatotic liver disease between patients with systemic lupus erythematosus (SLE) and controls. (**B**) Comparison of prevalence of significant fibrosis between patients with SLE and controls.

When comparing patients with SLE and controls, the controlled attenuation parameter [230 dB/m (IQR 192–266) vs 264 dB/m (IQR 208–306), p < 0.001] and prevalence of SLD (21.7% vs 41.5%, p < 0.001), were significantly lower in patients with SLE. On the other hand, LSM-TE [4.1 kPa (IQR 3.6–5.1) vs 4.0 kPa (IQR 3.4–5.0), p = 0.15] and the prevalence of significant fibrosis (6.6% vs 2.4%, p = 0.06) were not different between the groups (Figs. 1 and 2). Patients with SLE had a higher prevalence of arterial hypertension (24% vs 4.7%, p < 0.001), higher triglyceride levels [129 mg/dl (IQR 95–180) vs 114 mg/dl (IQR 85.8–162.5), p = 0.04], lower low-density lipoprotein cholesterol levels [97 mg/dl (IQR 79–116) vs 110.5 mg/dl (IQR 85–129), p = 0.009], and lower alanine aminotransferase [17.2 U/L (IQR 12.2–26.7) vs 20.3 U/L (14.6–29.4), p = 0.01], and also a numerically higher frequency of diabetes (6.6% vs 1.9%, p = 0.05). The remaining characteristics are presented in Table 1.

**Table 1:** Comparison of clinical and laboratory data between study groups.

	Total participants n = 318	SLE n = 106	Controls n = 212	p value
Clinical				
Age, years (IQR)	42 (30–52)	37 (29–51)	44 (30–52)	0.07
Female participants, n (%)	267 (84.0)	89 (84.0)	178 (84.0)	1.0
Weight, Kg (IQR)	66.9 (58.3–77.9)	66.5 (57.9–79.8)	67.1 (58.4–78.9)	0.71
BMI, (IQR)	26.6 (23.3–30.0)	26.3 (22.9–29.9)	26.7 (23.5–30.2)	0.36
Waist, cm (IQR)	88 (78.5–96.0)	89 (79.3–96)	87 (78–96)	0.38
Comorbidities, n (%)				
Diabetes	11 (3.5)	7 (6.6)	4 (1.9)	0.05
Obesity	80 (25.2)	25 (23.6)	55 (25.9)	0.65
Hypertension	36 (11.3)	26 (24.5)	10 (4.7)	< 0.0001
Hypertriglyceridemia	34 (10.7)	34 (32.1)	0 (0)	< 0.0001
Laboratory parameters				
Glucose, mg/dL (IQR)	88 (82–93)	84 (79–92)	88 (84–94)	< 0.0001
Creatinine, mg/dL (IQR)	0.72 (0.62–0.83)	0.76 (0.60–0.88)	0.72 (0.62–0.82)	0.27
Total cholesterol, mg/dL (IQR)	177 (151–204.5)	167.5 (138–197.8)	179 (154–208)	0.03
HDL, mg/dL (IQR)	48 (40–58)	45 (38–56)	49 (41–58)	0.01
LDL, mg/dL (IQR)	107 (83.5–126.5)	97 (79–116)	110.5 (85–129)	0.009
Triglycerides, mg/dL (IQR)	120 (89–169)	129 (95–180)	114 (85.8–162.5)	0.04
Total bilirubin, mg/dL (IQR)	0.55 (0.43–0.74)	0.48 (0.36–0.65)	0.60 (0.46–0.76)	< 0.0001
AST, U/L (IQR)	19.3 (16.2–24.4)	19.3 (14.85–24.4)	19.3 (16.7–24.4)	0.51
ALT, U/L (IQR)	18.7 (13.8–27.7)	17.2 (12.2–26.7)	20.3 (14.6–29.4)	0.01
Albumin, g/dL (IQR)	4.3 (4.05–4.53)	4.04 (3.71–4.33)	4.38 (4.19–4.59)	< 0.0001
Hemoglobin, g/dL (IQR)	14.3 (13.5–15.2)	13.8 (12.6–14.9)	14.7 (13.9–15.4)	< 0.0001
Platelets, cells × 10^9^/L (IQR)	249 (205–290)	231 (182–279.3)	257 (215–295)	0.001
Leukocytes, cells × 10^9^/L (IQR)	5.7 (4.7–6.7)	4.8 (3.9–6.3)	5.9 (5.1–6.8)	< 0.0001
Neutrophils, cells × 10^9^/L (IQR)	3.34 (2.64–4.23)	3.04 (2.38–4.30)	3.47 (2.76–4.22)	0.02
Lymphocytes, cells × 10^9^/L (IQR)	1.75 (1.33–2.14)	1.22 (0.79–1.75)	1.88 (1.59–2.23)	< 0.0001
C-reactive protein, mg/dL (IQR)	0.20 (0.10–0.44)	0.23 (0.11–0.63)	0.19 (0.10–0.36)	0.08

ALT: alanine aminotransferase; AST: aspartate aminotransferase; BMI: body mass index; CAP: controlled attenuation parameter; HDL: high-density lipoprotein; IQR: interquartile range; LDL: low-density lipoprotein; SLE: systemic lupus erythematosus.

### Comparison between patients with SLE with and without steatotic liver disease

Regarding components associated with metabolic syndrome, patients with SLD had higher BMI [31 (27–34) vs 25 (22–29), p < 0.001], glucose [88.5 mg/dl (IQR 82–93) vs 83.5 mg/dl (IQR 78–90), p = 0.03], and triglycerides [142 mg/dl (IQR 110–208) vs 123 mg/dl (IQR 95–172), p = 0.04]. However, the prevalences of diabetes and hypertension did not differ. Significant fibrosis was more common in patients with SLD (17% vs 4%, p = 0.02). In terms of variables of interest in patients with SLE, patients with SLD had a lower frequency of hydroxychloroquine use (65% vs 84%, p = 0.04), and higher C3 levels [123 mg/dl (IQR 102–136) vs 99 mg/dl (IQR 78–121), p = 0.004]. The remaining characteristics are presented in Table 2.

**Table 2:** Comparison between systemic lupus erythematosus patients with and without steatosis.

	Total n = 106	No steatosis n = 83	Steatosis n = 23	´p value
Clinical				
Female participants, n (%)	89 (84)	72 (87%)	17 (74%)	0.1
Age, years (IQR)	37 (29–51)	38 (29–51)	34 (30–55)	0.8
Weight, kg (IQR)	66.5 (58–76.7)	63 (57–73)	77 (68–89)	< 0.001
BMI, (IQR)	26.3 (22.9–29.9)	25 (22–29)	31 (27–34)	< 0.001
BMI ≥ 25, n (%)	60 (57)	41 (49%)	19 (83%)	0.004
BMI ≥ 30, n (%)	25 (24)	13 (16%)	12 (52%)	< 0.001
Waist, cm (IQR)	89 (79.5–96)	87 (77–93)	102 (93–108)	< 0.001
Prediabetes, n (%)	8 (8)	6 (7%)	2 (9%)	0.8
Diabetes, n (%)	7 (7)	4 (5%)	3 (13%)	0.2
Hypertension, n (%)	26 (24)	20 (24%)	6 (26%)	0.8
Hypertriglyceridemia, n (%)	33 (38)	26 (38%)	7 (41%)	0.8
Hypothyroidism, n (%)	18 (17)	15 (18%)	3 (13%)	0.6
LSM-TE results				
Stifness, kPa (IQR)	4.1 (3.6–5.1)	4 (3.6–4.9)	4.6 (3.4–7.2)	0.09
Significant fibrosis, n (%)	7 (7)	3 (4%)	4 (17%)	0.02
SLE-associated variables				
Time since diagnosis, years (IQR)	7.5 (4.5–14.0)	7 (4.5–14)	8 (4–16)	0.5
Current prednisone use, n (%)	51 (48)	41 (49%)	10 (43%)	0.5
Current prednisone dose > 5 mg, n (%)	25/51 (49)	17/39 (44%)	8/12 (67%)	0.09
Cumulative use of glucocorticoids, mg (IQR)	144,400 (9750–22,575)	14,400 (9450–21,825)	17,400 (11,025–30,225)	0.2
Current methotrexate use, n (%)	12 (11)	10 (12%)	2 (9%)	0.6
Current hydroxychloroquine use, n (%)	85 (80)	70 (84%)	15 (65%)	0.04
SLICC/ACR-DI, points (IQR)	1 (0–1)	1 (0–1)	1 (0–1)	0.8
SLEDAI-2K, points (IQR)	4 (2–6)	4 (2–6)	2 (1–6)	0.4
Laboratory parameters				
Glucose, mg/dl (IQR)	84 (79–92)	83.5 (78–90)	88.5 (82–93)	0.03
Creatinine, mg/dl (IQR)	0.76 (0.60–0.85)	0.76 (0.6–0.9)	0.69 (0.58–0.84)	0.3
Total colesterol, mg/dl (IQR)	174 (138–198)	174 (136–198)	167 (154–199)	0.3
HDL, mg/dl (IQR)	46 (38–57)	47 (38–62)	39 (35–46)	0.07
LDL, mg/dl (IQR)	97.5 (80.5–116)	95 (79–116)	114 (90–133)	0.7
Triglycerides, mg/dl (IQR)	129.5 (97–179)	123 (95–172)	142 (110–208)	0.04
Thyroid stimulating hormone, mIU/L (IQR)	2.54 (1.87–4.87)	2.5 (1.7–4.7)	3.6 (2.4–7.0)	1.0
Total bilirrubin, mg/dl (IQR)	0.48 (0.36–0.65)	0.48 (0.36–0.65)	0.5 (0.39–0.66)	0.8
Alkaline phosphatase, U/L (IQR)	72.5 (62–84.5)	71 (62–82)	77 (58–90)	0.8
AST, U/L (IQR)	19.4 (14.9–24.3)	19 (15–24)	20 (18–29)	0.09
ALT, U/L (IQR)	17.2 (12.2–24.3)	17 (12–23)	19 (15–28)	0.06
Albumin, g/dl (IQR)	4.0 (3.7–4.3)	4.1 (3.7–4.3)	4 (3.8–4.4)	1.0
Hemoglobin, g/dl (IQR)	13.8 (12.6–14.9)	13.6 (12.5–14.6)	14.2 (13.8–15.5)	0.02
Platelets, cells × 10^9^/L (IQR)	231 (182–278)	232 (182–285)	228 (182–266)	0.4
Leukocytes, cells × 10^9^/L (IQR)	4.8 (4.0–6.2)	4.6 (3.9–6)	5.7 (4.4–7.9)	0.02
Neutrophils, cells × 10^9^/L (IQR)	3.0 (2.4–4.3)	3 (2.4–3.6)	3.4 (2.2–5.3)	0.02
Lymphocytes, cells × 10^9^/L (IQR)	1.2 (0.8–1.7)	1.2 (0.7–1.7)	1.2 (1.0–1.9)	0.1
Neutrophil lymphocyte ratio, median (IQR)	2.7 (1.7–4.3)	2.6 (1.7–4.1)	2.9 (2.1–4.4)	0.6
C-reacctive protein, mg/dl (IQR)	0.21 (0.10–0.58)	0.22 (0.11–0.55)	0.18 (0.09–74)	0.5
C3, mg/dl (IQR)	102 (81–124)	99 (78–121)	123 (102–136)	0.004
C4, mg/dl (IQR)	17 (12–25)	16 (12–25)	20 (14–25)	0.3

ALT: alanine aminotransferase; AST: aspartate aminotransferase; BMI: body mass index; CAP: controlled attenuation parameter; HDL: high-density lipoprotein; IQR: interquartile range; LDL: low-density lipoprotein; SLE: systemic lupus erythematosus; SLEDAI-2K: Systemic Lupus Erythematosus Disease Activity Index 2000; SLICC/ACR-DI: Systemic Lupus International Collaborating Clinics / American College of Rheumatology Damage Index.

### Variables associated with steatotic liver disease and significant fibrosis

In the univariate analysis of SLE patients, the factors associated with SLD were BMI, waist circumference, BMI ≥ 30, glucose, and C3 levels. Hydroxychloroquine use was also associated, but as a protective factor (Table 3). In the case of controls, the variables that were associated with SLD in univariate analysis were age, BMI, waist circumference, BMI ≥ 30, glucose, triglycerides, high-density cholesterol, low-density cholesterol, and leukocytes (Table 3). In terms of significant fibrosis, the variables that were associated in univariate analysis in SLE patients were BMI, waist circumference, obesity, triglycerides, high-density cholesterol, low-density cholesterol, C4 levels, and C-reactive protein. In the case of controls, diabetes was the only variable associated with fibrosis.

**Table 3:** Variables associated with steatosis and significant fibrosis in SLE patients and controls.

	Steatosis				Fibrosis			
	SLE patients		Controls		SLE patients		Controls	
	OR (95% CI)	p	OR (95% CI)	p	OR (95% CI)	p	OR (95% CI)	p
Clinical data								
Age, years	0.99 (0.97–1.03)	0.85	1.03 (1.01–1.05)	0.006	0.89 (0.94–1.05)	0.89	1.07 (0.99–1.15)	0.10
BMI, kg/m^2^	1.25 (1.11–1.39)	< 0.0001	1.37 (1.25–1.50)	< 0.0001	1.24 (1.06–1.44)	0.005	1.10 (0.93–1.29)	0.29
Waist circumference, cm	1.13 (1.07–1.20)	< 0.0001	1.14 (1.09–1.18)	< 0.0001	1.08 (1.00–1.15)	0.04	1.02 (0.94–1.10)	0.63
Nutritional status								
Normal weight (BMI < 25)	Reference		Reference		Reference		Reference	
Overweight (BMI ≥ 25)	2.96 (0.79–11.09)	0.11	5.96 (2.62–13.5)	< 0.0001	Not estimable		1.90 (0.17–21.37)	0.60
Obesity (BMI ≥ 30)	10.15 (2.79–36.87)	< 0.0001	26.67 (10.36–68.65)	< 0.0001	5.75 (1.03–32.17)	0.05	2.83 (0.25–30.02)	0.40
Diabetes	2.96 (0.61–14.31)	0.2	4.34 (0.44–42.44)	0.21	2.58 (0.26–25.06)	0.4	17.00 (1.44–200.99)	0.03
Hypertension	1.11 (0.38–3.20)	0.8	2.19 (0.60–8.02)	0.23	2.48 (0.51–11.89)	0.2	Not estimable	
Laboratory parameters								
Glucose, mg/dl	1.04 (1.00–1.07)	0.044	1.08 (1.05–1.12)	< 0.0001	0.97 (0.89–1.04)	0.4	1.01 (0.99–1.03)	0.16
Triglycerides, mg/dl	1.00 (0.99–1.01)	0.09	1.01 (1.01–1.02)	< 0.0001	0.93 (0.87–0.99)	0.02	1.00 (0.99–1.01)	0.99
HDL, mg/dl	0.96 (0.92–1.00)	0.08	0.93 (0.89–0.95)	< 0.0001	0.86 (0.75–0.97)	0.02	0.98 (0.91–1.06)	0.63
LDL, mg/dl	1.00 (0.99–1.01)	0.7	1.01 (1.00–1.02)	0.009	0.97 (0.94–0.99)	0.05	0.99 (0.96–1.03)	0.76
Leukocytes, cells × 10^9^/L	1.22 (0.99–1.50)	0.06	1.34 (1.09–1.64)	0.005	0.72 (0.42–1.23)	0.2	1.38 (0.81–2.37)	0.24
C-reactive protein, mg/dl	1.25 (0.62–2.50)	0.5	1.18 (0.95–1.47)	0.13	2.77 (1.20–6.37)	0.02	1.17 (0.74–1.85)	0.51
C3, mg/dl	1.03 (1.01–1.05)	0.006	–	–	0.98 (0.95–1.00)	0.09	–	–
C4, mg/dl	1.02 (0.96–1.07)	0.58	–	–	0.88 (0.78–0.99)	0.05	–	–
SLE-associated variables								
Time since diagnosis, years	1.02 (0.96–1.07)	0.5	–		0.94 (0.82–1.08)	0.4	–	–
Prednisone use	0.79 (0.31–1.99)	0.6	–		0.79 (0.16–3.74)	0.7	–	–
Hydroxychloroquine use	0.34 (0.12–0.99)	0.05	–		Not estimable		–	–
Methotrexate use	0.69 (0.14–3.42)	0.6	–		1.33 (0.14–12.13)	0.8	–	–
Cumulative use of glucocorticoids, mg	1.00 (0.99–1.00)	0.17	–		1.00 (0.99–1.00)	0.8	–	–
SLEDAI-2K, points	0.95 (0.84–1.06)	0.4	–		1.08 (0.92–1.25)	0.3	–	–
SLICC/ACR-DI, points	1.06 (0.69–1.62)	0.8	–		1.13 (0.58–2.19)	0.7	–	–

BMI: body mass index; CI: confidence interval; HDL: high-density lipoprotein; LDL: low-density lipoprotein; OR: odds ratio; SLE: systemic lupus erythematosus; SLEDAI-2K: Systemic Lupus Erythematosus Disease Activity Index 2000; SLICC/ACR-DI: Systemic Lupus International Collaborating Clinics/American College of Rheumatology Damage Index.

To determine whether SLE was associated with SLD and/or significant fibrosis, we conducted a logistic regression analysis considering the whole sample (n = 318). On univariate analysis, SLE was found to be a protective factor against SLD (OR 0.39, 95% CI 0.23–0.67), but after adjusting for age, BMI, waist circumference, glucose, triglycerides, high-density cholesterol, low-density cholesterol, leucocytes, and hydroxychloroquine use, SLE was no longer associated with SLD (OR 0.43, 95% CI 0.10–1.91) (Supporting Information Table 1). SLE also showed no association with significant fibrosis (OR 2.93, 95% CI 0.91–9.45).

## Discussion

In this cross-sectional study, we aimed to assess whether SLE was associated with SLD and to determine which factors were associated with SLD and significant fibrosis. After multivariate adjustment, we found that SLE was not associated with SLD. The factors associated with SLD were anthropometric data, glucose, hydroxychloroquine, and C3 levels; meanwhile, anthropometric data, triglycerides, high-density lipoprotein, low-density lipoprotein, C4 levels, and C-reactive protein levels were associated with fibrosis.

Patients with SLE, an IMID, are known to have an increased prevalence of metabolic syndrome^9^, but this does not seem to be the case for SLD, which is considered to be the hepatic manifestation of metabolic syndrome. In this study, we found a 22% prevalence of SLD in a group of patients with SLE. In contrast to what has been found in other IMIDs^21,22^, the prevalence of SLD was lower in patients with SLE than in the general population (21.7% vs 41.5%). The connection between inflammatory diseases like IMIDs and metabolic syndrome, including SLD, isn't entirely clear. However, it's believed that ongoing inflammation may disrupt certain processes in the body, such as cytokine and adipokine regulation, leading to insulin resistance and SLD^23^. Therefore, we suspect that patients in remission, devoid of ongoing inflammation like those in our study, might have a lower risk of SLD compared to those with active disease. This, coupled with the younger age of our SLE patients, could partially explain the low prevalence of SLD. Our results are in line with the 23% prevalence reported by Yetginoglu et al. in 60 patients with SLE^11^. Of note, in that study, the CAP cutoff point that was used to define steatosis was 238 dB/m rather than the more strongly recommended 275 dB/m, so it is possible that the prevalence in their study could even had been overestimated. In contrast, the prevalence of significant fibrosis was lower in our study, but there was no operational definition of fibrosis in the study by Yetginoglu et al., so we cannot make any assumptions. Our results are also similar to those found in a recent meta-analysis of 2178 patients with rheumatoid arthritis, which showed a prevalence of SLD of 35.3%, which was similar to that described in the general population^7^. According to this, we cannot assume that the risk of SLD is shared equally between the different IMIDs.

Regarding factors associated with SLD that we found in our study, it is not surprising that metabolic factors such as BMI, waist circumference, and glucose levels were associated with SLD. This is similar to what Yetginoglu et al. found in their study^11^, and also to what Zamani et al. found in the meta-analysis on rheumatoid arthritis mentioned above, where BMI, rather than methotrexate, was associated with SLD^7^. In contrast, similar to what Yetginoglu et al. reported in their study, steroid use was not found to be associated with SLD^11^. It is worth emphasizing that even though patients with SLE traditionally have a higher prevalence of metabolic comorbidity (i.e. diabetes, hypertension, hypertriglyceridemia, and hypoalphalipoproteinemia), which has been described by other authors^9^, they did not have a higher prevalence of SLD in this study. A potential explanation is hydroxychloroquine, which was found to be a protective factor. This finding does not seem to be a spurious association, as hydroxychloroquine has been associated with a reduced risk of diabetes and metabolic syndrome in patients with rheumatoid arthritis^24,25^ and SLE^10,26,27^, most probably due to an effect on insulin sensitivity, and increased beta cell function, as well as anti-inflammatory and anti-oxidant mechanisms^28^. Therefore, this medication may contribute to the absence of a higher rate of SLD despite an increased prevalence of other metabolic conditions among SLE patients. Additionally, SLE patients exhibited a comparatively better metabolic profile, characterized by lower glucose and low-density lipoprotein levels. This improvement may result from the diligent monitoring and regular clinic visits that SLE patients typically undergo. Moreover, although age differences between SLE patients and controls did not reach statistical significance, the younger age of SLE patients might also play a role in the observed lower prevalence of steatosis. Considering these findings, universal screening for SLD in SLE patients may not be warranted. Instead, a tailored approach based on individual risk factors, similar to recommendations for the general population, would be more appropriate^29^.

Our study has limitations, including its cross-sectional nature and that it was conducted in a single center study with participants who were mostly in clinical remission. In addition, waist circumference measurement in SLE patients was not standardized. One major limitation is the selection of controls. Because they were from a separate study with different objectives, they did not share the same selection criteria, such as limiting alcohol intake equal to or less than 20–30 g per day. Nonetheless, 90% of the controls reported a weekly alcohol intake equal to or less than 30 g, and 100% reported a weekly intake equal to or less than 100 g, making alcohol unlikely as a significant cause of steatosis in controls. Also, based on their low prevalence of hypertension, diabetes, obesity, and SLD, and the inclusion criteria considering only well-controlled diabetes and hypertension, our study participants do not seem to reflect the general population, as they appear to be somewhat healthier. However, it's noteworthy that our study's main finding is the association of SLE with a lower prevalence of SLD, rather than a higher one. Thus, even if our controls had a higher prevalence of SLD, it wouldn't have altered our conclusions. Moreover, aside from alcohol, the exclusion of other liver diseases was done on clinical grounds, but no studies were performed to rule out other conditions (*e.g.* viral hepatitis, autoimmune hepatitis, hemochromatosis, Wilson´s disease, celiac disease, and autoimmune liver diseases). Another significant limitation of our study is the omission of data on azathioprine, mycophenolate, and other less commonly used agents such as rituximab or belimumab. These medications were not included in our study database because they are not typically linked to fibrosis or liver steatosis, the primary outcome of our research. However, we recognize that including this information would have been essential for a comprehensive description of patients with SLE. Azathioprine, for instance, is mainly linked to rare cases of non-cirrhotic portal hypertension and acute drug-induced liver injury with jaundice, which wouldn't likely affect our findings. Another limitation is that the diagnosis of SLD was based on the CAP, and not on the gold standard, which is the liver biopsy. Finally, due to the limited sample size and low prevalence of SLD in patients with SLE, we could not conduct a multivariate analysis in this population.

In conclusion, our study suggests that the prevalence of SLD in patients with SLE and low disease activity does not appear to be higher than that in the general population, despite the higher frequency of metabolic conditions such as diabetes, hypertension, and dyslipidemia among SLE patients. The potential role of hydroxychloroquine in preventing and treating metabolic syndrome, including SLD, in patients with IMIDs, warrants further investigation.

### Supplementary Information

Supplementary Table 1.

## Data Availability

Derived data supporting the findings of this study are available from the corresponding author [CMV] on request.
