# Supplementary material for: A cross sectional study assessing steatotic liver disease in patients with systemic lupus erythematosus
Source: Sci Rep. 2024 Jun 20;14:14275. doi: 10.1038/s41598-024-65105-1 (PMC11190197; doi:10.1038/s41598-024-65105-1)
Supplement: Supplementary file 1 — Supplementary Table 1. [file 41598_2024_65105_MOESM1_ESM.docx]

A cross sectional study assessing steatotic liver disease in patients with systemic lupus erythematosus

| Supporting Information Table 1.  Multivariable regression model to determine the association of SLE with steatotic liver disease. | | | |
| --- | --- | --- | --- |
|  | Odds Ratio | 95% confidence interval | p Value |
| Lupus | 0.43 | 0.10-1.91 | 0.27 |
| Age, years | 0.99 | 0.97-1.03 | 0.78 |
| BMI, kg/m^2^ | 1.14 | 0.99-1.3 | 0.06 |
| Waist circunference, cm | 1.07 | 1.01-1.13 | 0.04 |
| Glucose, mg/dl | 1.03 | 1.00-1.01 | 0.05 |
| Triglycerides, mg/dl | 1.00 | 0.99-1.00 | 0.26 |
| HDL, mg/dl | 0.96 | 0.93-0.99 | 0.02 |
| LDL, mg/dl | 1.00 | 0.99-1.01 | 0.47 |
| Leucocytes, cells x10^9^/L | 1.09 | 0.90-1.32 | 0.40 |
| Hydroxychloroquine use | 0.36 | 0.06-1.98 | 0.24 |
| BMI: body mass index; CI: confidence interval; HDL: high-density lipoprotein; LDL: low-density lipoprotein; SLE: systemic lupus erythematosus | | | |
